# YcfD_RM_ is a thermophilic oxygen-dependent ribosomal protein uL16 oxygenase

**DOI:** 10.1007/s00792-018-1016-9

**Published:** 2018-03-09

**Authors:** Rok Sekirnik, Sarah E. Wilkins, Jacob Bush, Hanna Tarhonskaya, Martin Münzel, Aayan Hussein, Emily Flashman, Shabaz Mohammed, Michael A. McDonough, Christoph Loenarz, Christopher J. Schofield

**Affiliations:** 1 Chemistry Research Laboratory, Department of Chemistry, University of Oxford, 12 Mansfield Road, Oxford, OX1 3TA UK; 2 Present Address: Novartis Technical Operations, Biosimilars, BTDM Mengeš, Lek d.d., Kolodvorska 27, 1234 Mengeš, Slovenia; 3 Present Address: AbbVie Deutschland GmbH and Co. KG, Drug Product Development, Knollstraßem, 67061 Ludwigshafen, Germany; 4 Present Address: Novo Nordisk A/S, Novo Nordisk Park, 2760 Måløv, Denmark; 5 Present Address: Institute of Pharmaceutical Sciences, Albert-Ludwigs-Universität Freiburg, 79104 Freiburg, Germany

**Keywords:** Ribosomes, Translation, Protein synthesis, Hydroxylase, Dioxygenase, Post-translational modification, Oxygen/hypoxia sensing, Thermophilic enzyme

## Abstract

YcfD from *Escherichia coli* is a homologue of the human ribosomal oxygenases NO66 and MINA53, which catalyse histidyl-hydroxylation of the 60S subunit and affect cellular proliferation (Ge et al., Nat Chem Biol 12:960–962, 2012). Bioinformatic analysis identified a potential homologue of ycfD in the thermophilic bacterium *Rhodothermus marinus* (ycfD_RM_). We describe studies on the characterization of ycfD_RM_, which is a functional 2OG oxygenase catalysing (*2S*,*3R)*-hydroxylation of the ribosomal protein uL16 at R82, and which is active at significantly higher temperatures than previously reported for any other 2OG oxygenase. Recombinant ycfD_RM_ manifests high thermostability (*T*_m_ 84 °C) and activity at higher temperatures (*T*_opt_ 55 °C) than ycfD_EC_ (*T*_m_ 50.6 °C, *T*_opt_ 40 °C). Mass spectrometric studies on purified *R.* *marinus* ribosomal proteins demonstrate a temperature-dependent variation in uL16 hydroxylation. Kinetic studies of oxygen dependence suggest that dioxygen availability can be a limiting factor for ycfD_RM_ catalysis at high temperatures, consistent with incomplete uL16 hydroxylation observed in *R.* *marinus* cells. Overall, the results that extend the known range of ribosomal hydroxylation, reveal the potential for ycfD-catalysed hydroxylation to be regulated by temperature/dioxygen availability, and that thermophilic 2OG oxygenases are of interest from a biocatalytic perspective.

**Electronic supplementary material:**

The online version of this article (10.1007/s00792-018-1016-9) contains supplementary material, which is available to authorized users.

## Introduction

Ferrous iron and 2-oxoglutarate (2OG)-dependent oxygenases (2OG oxygenases) are a ubiquitous enzyme superfamily that catalyses a wide range of oxidative reactions (Hausinger and Schofield 2015). Their biochemical roles are diverse and include the catalysis of steps in secondary metabolite biosynthesis in plants and microbes and the regulation of transcription in most, if not all, eukaryotes. In humans and other animals, 2OG oxygenases play a central role in coordinating the cellular and physiological responses to limiting oxygen levels (hypoxia) (Hausinger and Schofield 2015; Schofield and Ratcliffe 2004). In humans, the cellular activity of the transcription factor hypoxia inducible factor (HIF) prolyl-hydroxylases (PHDs or EGLNs) is limited by O_2_ availability; together with other properties, this is proposed to enable the PHDs to act as hypoxia sensors for the important HIF system (Ehrismann et al. 2007). HIF activity is also regulated via asparaginyl hydroxylation as catalysed by factor inhibiting HIF (FIH), which belongs to the Jumonji C (JmjC) family of 2OG oxygenases (Hewitson et al. 2003). However, the HIF pathway appears to be limited to animals (Loenarz et al. 2011); in microbes, other well-characterised mechanisms not involving 2OG oxygenases are established as hypoxia sensors (Taabazuing et al. 2014).

Intrigued by the similarity of FIH to apparent bacterial JmjC proteins, we recently identified *Escherichia coli* ycfD (ycfD_EC_) as an arginyl hydroxylase, which catalyses hydroxylation of R81 (homologous to R82 in *R. marinus*) in the *E. coli* 50S ribosomal protein uL16 (Ge et al. 2012) [ribosomal nomenclature is as according to Ban et al. (2014)]. R81 is located in the immediate vicinity of the peptidyl transferase centre and has been identified as one of the key uL16 residues that is involved in guiding A- to P-site tRNA translocation by facilitating ‘sliding’ and stepping’ mechanisms (Bock et al. 2013). YcfD_EC_ was first identified as a 2OG oxygenase that catalyses hydroxylation of a prokaryotic ribosomal protein, i.e. uL16 in *E. coli*, and is proposed to have functions relating to effects on global translation and growth rates under limiting nutrient conditions (Ge et al. 2012). Mina53 and NO66 are human homologues of ycfD_EC_. Mina53 and NO66 are not arginyl hydroxylases, instead being histidyl hydroxylases acting on eL27 and uL2, respectively (Ge et al. 2012). We have also identified hydroxylation of small ribosomal subunit uS12 and release factor eRF1 proteins as regulatory for the accuracy of protein synthesis (Feng et al. 2014; Katz et al. 2014; Loenarz et al. 2014; Singleton et al. 2014) in organisms ranging from yeasts to humans. Thus, the ribosomal oxygenases have to date been found in bacteria and eukaryotes, including humans, though they appear not to be present in archaea (Chowdhury et al. 2014).

YcfD appears to be highly conserved in Proteobacteria, in particular Gammaproteobacteria. Whilst mostly absent from other bacterial phyla, phylogenetic studies of ycfD homologues identified a small number of putative ycfD-like 2OG oxygenases in the phylum Bacteroidetes, in particular in species *Rhodothermus marinus* and *Salinibacter ruber* (Ge et al. 2012); no homologues were found in other thermophilic phyla. To our knowledge, no extremophilic 2OG oxygenase has previously been reported. We were, therefore, interested to observe evidence for a putative 2OG oxygenase with high sequence homology to ycfD in the genome of *R. marinus*, which is a thermo- and halophilic obligate aerobe that was first isolated from submarine alkaline hot springs in Iceland (Alfredsson et al. 1988). Reports suggest that *R.* *marinus* can only grow in a narrow zone in the hot springs, defined by temperature, salt concentration, content of organic material and O_2_ availability (Bjornsdottir et al. 2006). The optimal growth temperature for *R. marinus* of 65 °C is significantly higher than those for other organisms with characterised 2OG oxygenases. This suggested that the operating temperature range for 2OG oxygenases could extend to temperatures of at least 65 °C, at least for thermophile-based enzymes, with potential biotechnological implications. We also proposed that ycfD_RM_ may be more amenable to crystallographic analyses, in particular with respect to obtaining substrate complexes, than ycfD_EC_ (Chowdhury et al. 2014). Here, we report studies on the biochemical characterization of ycfD_RM_; the results reveal ycfD_RM_ is a *bona fide* 2OG oxygenase, the first characterised thermostable 2OG oxygenase. Furthermore, we propose that uL16 hydroxylation in cells may be limited by temperature and/or oxygen availability.

## Materials and methods

### Strains and growth conditions

*Rhodothermus marinus* (strain R-10 DSM 4252) was grown in ATCC Medium 1599 enhanced with NaCl (1% w/v) at 60–80 °C in an Innova44 (New Brunswick Scientific) shaking incubator either in unbaffled Pyrex^®^ Erlenmeyer flasks (2 L, filled to 0.6 L), or Tunair™ polypropylene flasks (2.5 L, filled to 1 L), shaken at 180 rpm.

When cultures reached an OD_600_ 0.9–1.0, the cells were harvested by centrifugation and stored at − 80 °C. The cell paste was lysed using glass beads in a PreCellys homogenizer (sonication was found to be insufficient for effective cell lysis). Ribosomal proteins were purified using a previously reported method (Hardy et al. 1969).

### LC–MS studies on ribosomal proteins

Ribosomal proteins were analysed by reverse-phase ultra-performance liquid chromatography (RP-UPLC; Waters BEH C4 reversed phase column, 2.1 × 150 mm, 1.7 μm particle size, 300 Å pore size) and electrospray ionisation time-of-flight mass spectrometry (ESI-TOF MS). Proteins were separated using a stepped gradient 0.1% formic acid in water to 0.1% formic acid in acetonitrile at 0.3 mL/min as described (Ge et al. 2012).

### Cloning of ycfD_RM_

*Rhodothermus marinus* genomic DNA was isolated using a DNA isolation kit (DNEasy; Qiagen). The gene of interest (gi: 345301926) was amplified from genomic DNA using the following primers: forward CACAGCCACGACGTTTACC; reverse GGAATCACGTTGACCCAGTC. Amplification of the gene by polymerase chain reaction was carried out over 40 cycles at 96 °C for 30 s, 64 °C for 30 s, and 72 °C for 75 s, using Bio-X-Act Long DNA Taq polymerase (Bioline Ltd). The PCR product was ligated into the pGEMT-Easy vector (Promega) from which the *ycfD*_*RM*_ gene was amplified with *NdeI*/*Sac*I restriction sites using the primers ATAAACATATGCAGCTTCCCGA/AGAATAGAGCTCAGCGTTTGC and sub-cloned into pET28a using the *Nde*I/*Sac*I restriction sites. YcfD_RM_ protein was expressed using the pET28a vector system in *E. coli* strain BL21 (DE3). Protein was purified using a HisTrap HP column (5 mL, GE Healthcare) followed by Superdex 75 size-exclusion column (300 mL, GE Healthcare) using 50 mM Tris–HCl, pH 7.5, 300 mM NaCl, and 5% glycerol as elution buffer.

### Enzymatic activity studies

Enzymatic activity was determined using a MALDI–MS-based assay. Unless indicated otherwise, the enzyme was incubated at 55 °C with (NH_4_)_2_Fe(SO_4_)_2_ (100 μM), sodium ascorbate (1 mM), 2OG (200 μM) and a peptide fragment of *R. marinus* uL16 (uL16_RM_) corresponding to residues ^72^KPVTKKPAEVRMGKGKGSVE^91^ (GL Biochem, Shanghai, China, 100 μM) in 50 mM HEPES buffer (pH 7.5). The reaction was quenched with an equal volume of 1% (v/v) aqueous CF_3_COOH_aq_ and analysed by MALDI–MS. Substrate turnover was defined as %OH = *I*_OH_/(*I*_OH_ + *I*_non-OH_) × 100%, where I_OH_ and I_non-OH_ correspond to peak intensities of hydroxylated and non-hydroxylated peptides, respectively.

To investigate the O_2_ dependence of the reaction, assays were performed at 37 °C in sealed vials which contained 500 µM peptide in the HEPES 50 mM, pH 7.5, buffer pre-equilibrated using mass-flow controllers (Brooks Instrument, UK) under N_2_/O_2_ gas mixes with variable O_2_ content (from 0 to 50%). Other components of the reaction were added using a Hamilton syringe prior to the assay, to final concentrations of 100 µM Fe(II), 1 mM l-ascorbate, 500 µM 2OG, 2 µM ycfD_RM_. The reaction was quenched with 1% (v/v) aqueous formic acid at defined time points. Substrate turnover was assessed by MALDI–TOF–MS as described above.

### LC–MS/MS assays

LC–MS/MS experiments were performed using an Orbitrap Elite machine (Thermo Fisher Scientific, DE, USA) connected to a UHPLC Proxeon EASY-nLC 1000 and an EASY-Spray nano-electrospray ion source. Peptides were trapped on an Acclaim PepMap^®^ trapping column (100 μm i.d. × 20 mm, 5 μm C18) and separated using an EASY-spray Acclaim PepMap^®^ analytical column (75 μm i.d. × 500 mm, RSLC C18, 2 μm, 100 Å). MS method parameters are detailed in Supplementary Information.

### Differential scanning fluorimetry (DSF) studies

Thermostability of the enzyme was measured by differential scanning fluorimetry (DSF) using a MiniOpticon Real-Time PCR Detection System (Bio-Rad). Fluorescence was measured between 4 and 95 °C, using FAM (492 nm) and RIOX (610 nm) excitation and emission filtres, respectively. The melting temperature (*T*_m_) is calculated using the Boltzmann equation $y = {\text{LL}} + \frac{{{\text{UL}} - {\text{LL}}}}{{1 + e^{{\frac{{T_{\text{m}} - x}}{a}}} }}$, where LL and UL are values of minimum and maximum intensities and *a* corresponds to the slope of the curve within *T*_m_.

### Circular dichroism (CD) studies

Circular dichroism measurements were acquired using a Chirascan CD spectrometer (Applied Photophysics) with a Peltier temperature-controlled cell holder. All experiments were performed in a 0.1-cm path length cuvette using 0.1–0.2 mg/mL protein in 10 mM sodium phosphate buffer (pH 8.0). Data were recorded from 240 to 185 nm, at 0.5 nm intervals, and each data point was averaged for 3 s. Spectra were base-line corrected and smoothed using the Savitzky–Golay filtre. Data recorded in the 185–240 nm range were analysed using DichroWeb and the CONTIN deconvolution method was used to estimate secondary structural content using reference set 6 (Provencher and Glockner 1981; Vanstokkum et al. 1990). In thermal denaturation experiments, spectra were recorded every 2–5 °C, with a 5-min equilibration time at each temperature. Temperature-dependent changes in secondary structure were monitored by CD at 218 nm, and normalised data fit to a Boltzmann sigmoidal curve in GraphPad Prism to determine *T*_m_ values. At the end of the melt, reversibility was determined by returning to the start temperature (10 °C) and comparing the CD spectrum with the spectrum obtained prior to denaturation.

### Amino acid analyses

Amino acid analyses were performed as previously described (Ge et al. 2012) with an enzymatic amino acid hydrolysis step performed as described (Feng et al. 2014).

## Results

**Fig. 1:**
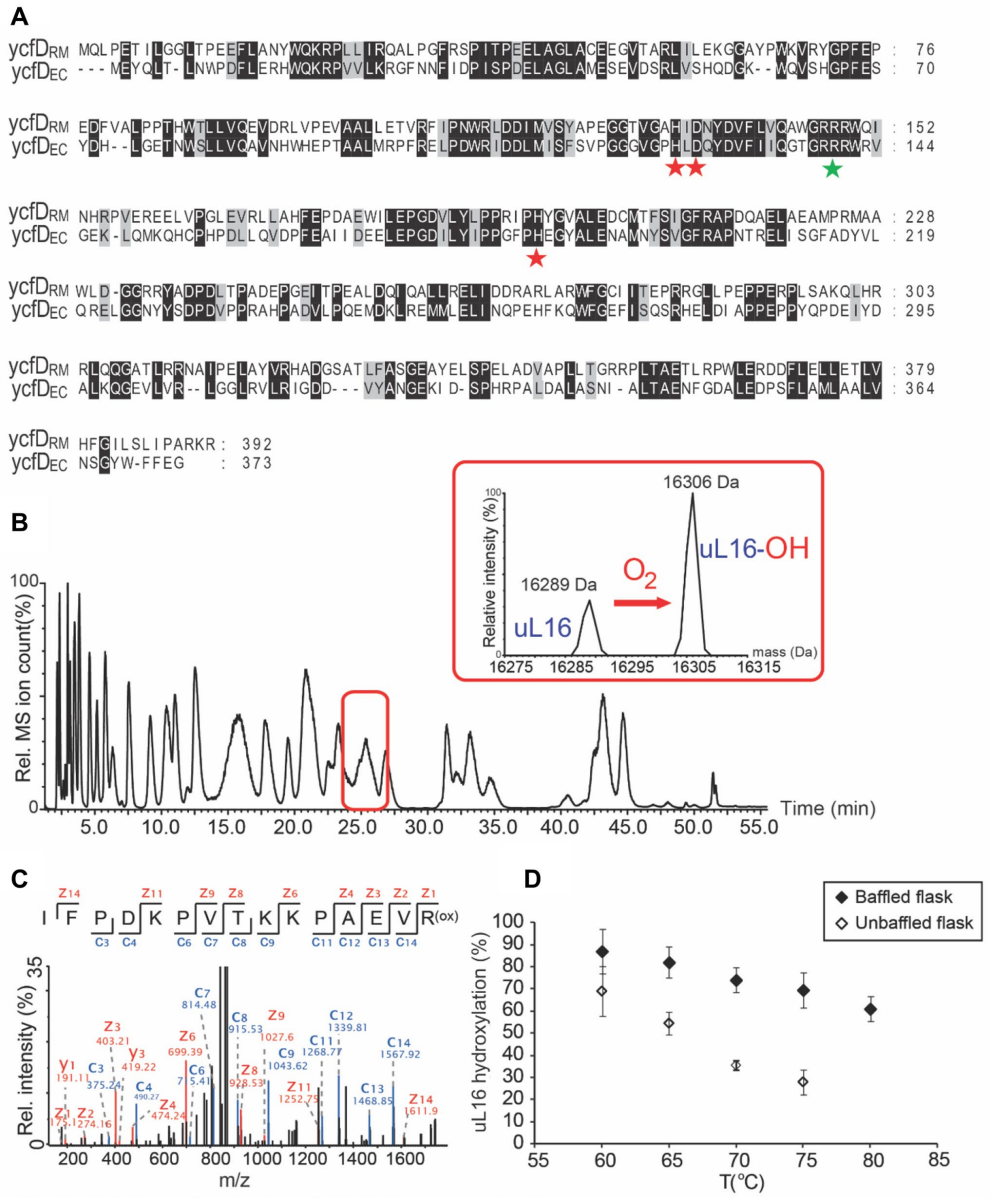
ycfD_RM_ from *R. marinus* catalyses oxygen-dependent ribosomal protein hydroxylation. **a** Protein sequence alignment of ycfD_RM_ and ycfD_EC_ produced with ClustalW and edited using GeneDoc 2.7. Red stars in **a** indicate the Fe(II)-binding facial triad and the green star indicates the basic residue that binds 2OG. **b** Ribosomal proteins were isolated by sucrose density centrifugation followed by acetone precipitation. UPLC protein separation coupled to ESI-TOF mass spectrometry was used to chromatographically separate and determine the intact masses of *R. marinus* ribosomal proteins. Inset: deconvoluted ESI–MS spectrum showing partial hydroxylation of ribosomal protein uL16_RM_. **c** MS/MS spectrum of uL16_RM_ fragment showing hydroxylation at R82; **d** Hydroxylation of uL16_RM_ decreases with an increase in growth temperature of *R. marinus* and varies for growth in baffled (2.5 L polypropylene filled to 1 L with media) and unbaffled flasks (2 L Pyrex filled to 0.6 L with media). uL16_RM_ intact protein masses were determined by ESI–MS spectrometry. Average of two independent MS experiments is shown with error bars denoting standard deviation of the mean. Deconvoluted MS spectra are shown in Supplementary Materials

Bioinformatic analysis of ycfD homologues (Ge et al. 2012) identified a homologous protein from the thermo- and halophilic organism *R.* *marinus* (gi: 345301926), ycfD_RM_. YcfD_RM_ has ~35% amino acid identity and ~55% similarity to ycfD from *E. coli* (ycfD_EC_, Fig. 1a). YcfD_RM_ residues corresponding to the ycfD_EC_ Fe(II)-coordinating triad are apparently conserved (ycfD_RM_ H133, D135, H196), as is the basic residue (R148) that is predicted to bind the 2OG C-5 carboxylate (Fig. 1a). Because ycfD_EC_ and its human homologues NO66 and MINA53 are ribosomal oxygenases, albeit with different target proteins and sequence selectivity (Ge et al. 2012), we proposed that ycfD_RM_ might also modify ribosomal proteins.

To study post-translational modifications of *R. marinus* ribosomal proteins, optimization of large-scale *R. marinus* growth was first conducted (see Supplementary Materials). Intact protein mass spectrometric analysis of *R.* *marinus* ribosomal proteins used a modification of our established UPLC–ESI–MS methodology (Ge et al. 2012); 48 out of 54 predicted ribosomal proteins in *R.* *marinus* were identified based on intact mass and their identity confirmed by MS/MS (Fig. 1 and Supplementary Tables 1 and 2). Intact masses of several other proteins from the 30S and the 50S ribosomal subunits were consistent with post-translational modifications, as described in Supplementary Materials (Supplementary Tables 1 and 2). Importantly, we observed two co-eluting species at retention time 25.3 min with masses 16,289 and 16,306 Da (Fig. 1b), consistent with unhydroxylated and hydroxylated uL16_RM_. uL16_RM_ is also subject to monomethylation, presumably on its *N*-terminal methionine as occurs in *E. coli* (Arnold and Reilly 1999). MS–MS analysis of trypsin-digested ribosomal proteins identified the hydroxylation site on R82 (Fig. 1c), analogous to the *E. coli* uL16 (uL16_EC_) R81, which is the hydroxylation substrate of ycfD_EC_. Interestingly, we observed incomplete uL16_RM_ hydroxylation (35%, Fig. 1d) at 70 °C, the optimal growth temperature of *R. marinus.* We cultured *R.* *marinus* between 60 and 75 °C and observed approximately linearly decreasing levels of uL16_RM_ hydroxylation. uL16_RM_ hydroxylation decreased from 60 to 25% as the growth temperature was increased from 60 to 75 °C. Hydroxylation of uL16_RM_ was higher in baffled flasks compared to unbaffled flasks (Fig. 1d), consistent with previous reports that baffled flasks provide adequate oxygen supplies for microbial growth and that oxygen consumption in bacterial cultures is substantially higher in baffled flasks compared to unbaffled flasks under similar growth conditions (McDaniel and Bailey 1969). Collectively, these observations imply that uL16_RM_ hydroxylation is sensitive to oxygen availability.

**Fig. 2:**
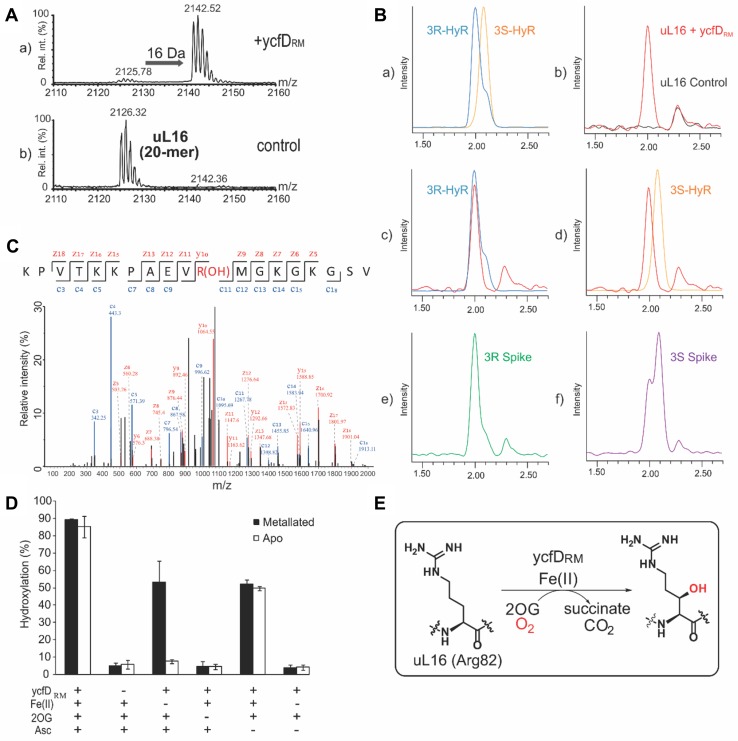
YcfD_RM_ is a 2-oxoglutarate-dependent oxygenase. **a** MALDI–MS spectrum of ycfD_RM_-dependent hydroxylation of *R. marinus* uL16_RM_ fragment (KPVTKKPAEVRMGKGKGSVE). A 16 Da mass shift is consistent with a ycfD_RM_-dependent oxidative modification. **b** Amino acid analysis reveals (2*S*, 3*R*)-hydroxylation of R82. Extracted ion chromatograms (*m*/*z* = 345) from LC–MS analysis of: *a* synthetic (2*S*, 3*S*)- and (2*S*, 3*R*)-3-hydroxy-arginine standards, *b*–*d* amino acid hydrolysates from ycfD_RM_-hydroxylated uL16_RM_ peptide fragment(red trace) overlaid with hydrolysates from a control peptide (black trace, *b*), (2*S*, 3*R*)-hydroxy-arginine standard (blue trace, *c*) or (2*S*, 3*S*)-hydroxy-arginine standard (yellow trace, *d*); *e*–*f* amino acid hydrolysates from ycfD_RM_-hydroxylated uL16_RM_ spiked with either (2*S*, 3*R*)-hydroxy-arginine (*e*) or (2*S*, 3*S*)-hydroxy-arginine (*f*) standards. **c** MS/MS studies on uL16_RM_ fragment peptide (KPVTKKPAEVRMGKGKGSVE-NH_2_) incubated with ycfD_RM_ and co-factors/co-substrates (Fe(II), 2OG and ascorbate) revealed hydroxylation at R82. **d** Co-factor dependence of ycfD_RM_:ycfD_RM_ (1 μM) was incubated in a reaction mixture from which co-factors and co-substrates (Fe(II), 2OG and ascorbate at 100 μM, 200 μM and 1 mM, respectively), were systematically removed. Apo- and metallated forms of ycfD_RM_ were tested. The reaction was carried out in 50 mM HEPES (pH 7.5) at 65 °C in triplicates. The mean value is shown, with error bars representing standard deviation. **e** Reaction scheme of ycfD_RM_-catalysed (2*S*, 3*R*)-arginine-3-hydroxylation

To investigate whether the observed hydroxylation of uL16_RM_ is indeed catalysed by ycfD_RM_, we then focused on the biochemical characterization of ycfD_RM_ in vitro. YcfD_RM_ was recombinantly produced in *E.* *coli* using Ni(II)-affinity chromatography and size-exclusion chromatography. Upon incubation of the uL16_RM_ peptide fragment (^76^KKPAEVRMGKGKGSVE^91^) with ycfD_RM_ and co-factors/co-substrates Fe(II) and 2OG, a + 16 Da mass shift, consistent with hydroxylation, was observed by MALDI–MS, confirming ycfD_RM_ as a *bona fide* 2OG oxygenase (Fig. 2a). MS/MS analysis of the hydroxylated peptide product confirmed the site of hydroxylation at residue R82 and amino acid analysis demonstrated (2*S,* 3*R*)-arginyl hydroxylation, thus having the same stereospecificity of hydroxylation as ycfD_EC_ (Fig. 2b, c), supporting the assignment of ycfD_RM_ as an arginyl hydroxylase of the ribosomal protein uL16_RM_. The catalytic activity of a ycfD_RM_ iron-binding variant H133A, predicted to abrogate binding of Fe(II), was reduced to background levels, suggesting that Fe(II) binding is essential for catalytic activity (Supplementary Figure 1).

**Fig. 3:**
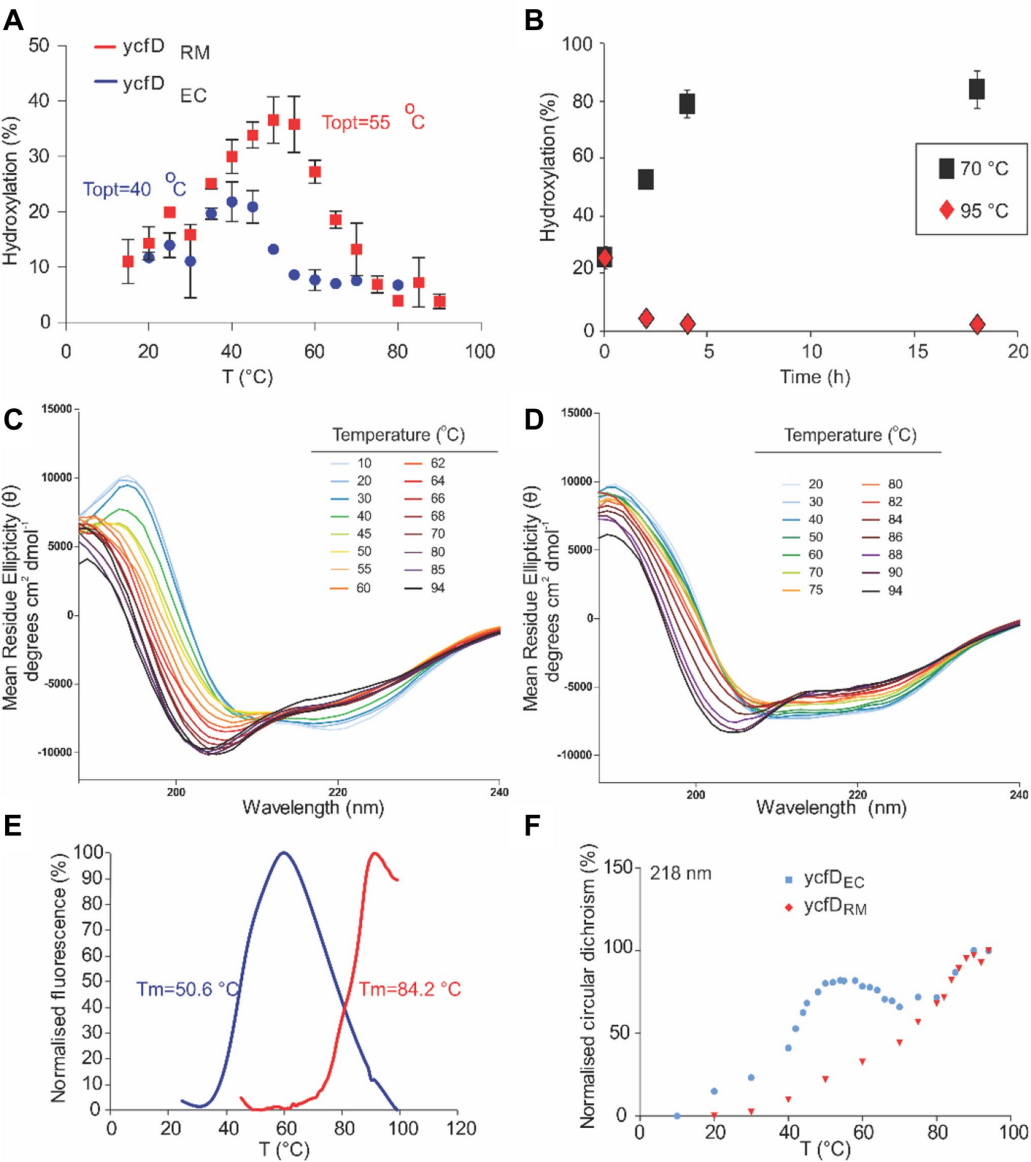
Temperature dependence of ycfD_EC_/ycfD_RM_ activity and stability. **a** ycfD_EC_ and ycfD_RM_ were incubated with *E. coli* and *R. marinus* uL16 fragment peptides, respectively, in the presence of co-factors/co-substrates (Fe(II), 2OG and ascorbate at 100 μM, 200 μM and 1 mM, respectively), in 50 mM HEPES, pH 7.5 (ycfD_RM_) or pH 8.0 (ycfD_EC_). Reaction mixtures were incubated under atmospheric conditions at the indicated temperatures before addition of the enzyme (1 μM) and reaction allowed to proceed for 3 min before quenching with equal volume of CF_3_COOHaq (1%). Substrate turnover was determined by MALDI–MS. Reactions were carried out in triplicate, with points indicating the average and error bars denoting standard deviation. **b** Results of pre-incubation of ycfD_RM_ at 70 °C (black squares) and 95 °C (red diamonds). Pre-incubation at 70 °C leads to increased activity; pre-incubation 95 °C leads to loss of catalytic activity. **c**, **d** Circular dichroism (CD) spectra of ycfD_RM_ and ycfD_EC_ between 20 and 94 °C. The CD signal was recorded between 190 and 260 nm while thermal denaturation was performed on ycfD_RM_ and ycfD_EC_ 20–94 °C. The signal at 218 nm was used to derive T_m_ values (**f**). A sigmoidal dose–response function was used to fit the data to derive the *T*_m_ value (GraphPad Prism, version 5.04, GraphPad Software). CD spectra of ycfD_RM_ and ycfD_EC_ thermal denaturation reveal a degree of denaturation above 80 °C with a retention of a significant degree of secondary structure up to 94 °C for ycfD_RM_, but not ycfD_EC_; **e** differential scanning fluorimetry (DSF) was used to investigate the temperature dependence of ycfD_EC_/ycfD_RM_ stability. Experiments were conducted in triplicate, with representative spectra shown

Next, we studied the biophysical properties of ycfD_RM_, compared to ycfD_EC_. We first studied the catalytic activity of ycfD_RM_ and ycfD_EC_ at different temperatures. YcfD_RM_ maintained activity at higher temperatures than ycfD_EC_, demonstrating maximum catalytic activity (*T*_opt_) at 55 °C, compared to 40 °C for ycfD_EC_ (Fig. 3a). YcfD_RM_ catalytic activity (measured at *T*_opt_) was retained after incubation at 70 °C for up to 18 h, whereas incubation at 95 °C led to complete inactivation (Fig. 3b). To compare the thermal stabilities of ycfD_RM_ and ycfD_EC_, their thermal denaturation was studied using circular dichroism (CD) spectroscopy. We monitored temperature-induced changes in CD at 218 nm (characteristic wavelength for beta-sheet) and determined *T*_m_ values of 41 and 85 °C for ycfD_EC_ and ycfD_RM_, respectively (Fig. 3c, d, f). Consistent with the CD measurements, an analogous study using differential scanning fluorimetry (DSF) demonstrated a similar difference in *T*_m_ values: 51 °C compared to 84 °C for ycfD_EC_ and ycfD_RM_, respectively (Fig. 3e).

While *T*_m_ values are thus comparable to the optimal growth temperature of *R. marinus*, the observed difference in optimal temperature (*T*_opt_) for catalysis by isolated recombinant ycfD_RM_ was > 15 °C below the optimal growth temperature of *R.* *marinus*. This observation may reflect non-optimal turnover conditions including the use of a fragment of the natural uL16_RM_ protein substrate, which may lead to enhanced inactivation at higher temperatures in vitro due to uncoupling of 2OG and substrate oxidation (Hausinger and Schofield 2015). Steady-state kinetic analysis at 37 °C revealed that *K*_m_^app^ values for Fe(II) and 2OG (14.6 ± 5.4 and 71.1 ± 12.2 μM, respectively) were likely not limiting for the activity of ycfD_RM_. Both values were found to be higher than for ycfD_EC_, for which *K*_m_^app^ values for Fe(II) and 2OG were 3.3 ± 1.4 and 8.6 ± 3.4 μM, respectively (Supplementary Figure 2).

Interestingly, the *K*_m_^app^ of 100 ± 20 μM O_2_ (Supplementary Figure 3) for ycfD_RM_ is in the range of O_2_ solubility at 70 °C [the calculated solubility of O_2_ at 70 °C and 3.5% (w/w) salinity is 128 μM according to the model of Tromans (1998)]. Oxygen availability could, therefore, limit the activity of ycfD_RM_ in cells, consistent with our LC–MS studies on purified *R. marinus* ribosomes. The *K*_m_^app^ for ycfD_EC_ was significantly lower than that for ycfD_RM_ (Supplementary Figure 3), suggesting that oxygen availability is less likely to be limiting for ycfD_EC_ activity, as is consistent with the observation of complete hydroxylation of uL16_EC_ as reported elsewhere (Arnold and Reilly 1999; Ge et al. 2012).

The ycfD target residue (R81 in *E. coli*, R82 in *R.* *marinus* and *T. thermophilus*) is located at the apex of a flexible loop that protrudes into the peptidyl transferase centre (PTC) in the 50S subunit (Voorhees et al. 2009). We, therefore, proposed that the activity of ycfD_RM_ with the short uL16_RM_ fragment substrate may be reduced due to conformational mobility of a linear peptide fragment. We synthesised a cyclic peptide mimic of the uL16_RM_ loop conformation to investigate whether a loop-like uL16_RM_ fragment is a better substrate for ycfD_RM_ (the synthesis is described in Supplementary Information). Guided by ribosome structures (when this work was carried out we did not have a ycfD substrate structure; Chowdhury et al. 2014), we designed a thioether-linked cyclic peptide (Supplementary Figure 4). In support of the subsequently obtained crystal structure for the ycfD_RM_–uL16_RM_ complex (Chowdhury et al. 2014), the cyclic peptide was more efficiently hydroxylated than the acyclic peptide by ycfD_RM_ (Supplementary Figure 4). Notably the *K*_m_^app^ for the synthetic cyclic uL16_RM_ peptide (^76^KKPAEVRMGKGKG^88^-linker) was 75 ± 23 μM, compared to *K*_m_^app^ of 208 ± 66 μM for the linear variant (Supplementary Figure 4). The k_cat_ value remained approximately constant (0.50 and 0.47 s^−1^ for linear and cyclic uL16 peptides, respectively), suggesting that the mechanism of hydroxylation is the same for the linear and cyclic variants, but that the pre-organisation of the cyclic peptide conformation better mimics the native conformation of uL16.

## Discussion

YcfD_EC_ is a 2OG- and Fe(II)-dependent oxygenase that catalyses arginyl hydroxylation of the ribosomal protein uL16_EC_, a modification linked to the overall rate of protein biosynthesis and cell growth (Ge et al. 2012). Human orthologues of ycfD, NO66 and MINA53 also catalyse ribosomal protein hydroxylation of eukaryotic ribosomal proteins from the large subunit, but of histidinyl rather than arginyl residues (Ge et al. 2012) (Chowdhury et al. 2014). The ycfD/NO66/MINA53 family is, therefore, evolutionarily and functionally conserved from prokaryotes to mammalians.

We identified a gene with high sequence similarity to ycfD_EC_ in *R. marinus* from the phylum Bacteroidetes. *R. marinus* is found in shallow alkaline hot springs where temperatures exceed 70 °C (Alfredsson et al. 1988). Given the diverse range of stereospecific oxidative modifications that 2OG oxygenases catalyse, including activation of unactivated C-H bonds (Hausinger and Schofield 2015; Martinez and Hausinger 2015), which is otherwise synthetically challenging (Hartwig 2016), their biocatalytic potential is substantial. The results presented here define ycfD_RM_ as a thermophilic 2OG oxygenase that, by analogy with ycfD_EC_ (Ge et al. 2012), likely functions as a hydroxylase of the ribosomal protein uL16_RM_. They thus expand the scope of ribosomal hydroxylation, and of 2OG oxygenase catalysis, to extremophilic organisms. The demonstration that it is possible to use purified thermostable recombinant 2OG oxygenases is of substantial interest from a biocatalytic perspective, in part because although (engineered) 2OG oxygenases are used for biocatalysis in cells, their lability appears to have precluded their widespread use in purified form.

Intact protein mass spectrometric analysis of ribosomal proteins from *R. marinus* revealed two species of 16289 ± 1 and 16306 ± 1 Da, consistent with masses of unhydroxylated and hydroxylated uL16_RM_. MS/MS analysis of uL16_RM_ identified the modification as hydroxylation of R82. We reasoned that the oxidative modification may be catalysed by the ycfD homologue identified in *R.* *marinus* genome. We cloned, expressed and purified ycfD_RM_ and studied its biochemical properties. YcfD_RM_ was found to be Fe(II), 2OG and O_2_ dependent, thus classifying it as a *bona fide* 2OG oxygenase.

Importantly, from the biocatalytic perspective, we demonstrated that ycfD_RM_ is more thermostable than ycfD_EC_ from *E. coli* (*T*_m_ of 84 vs. 51 °C) and, to our knowledge, any other characterised 2OG oxygenase, and with higher degree of catalytic turnover at 55–60 °C (compared to 45 °C for ycfD_EC_). The difference in *T*_m_ between ycfD_EC_ and ycfD_RM_ is characteristic of thermophilic adaptation of protein structure. Thermophilic proteins are frequently adapted for stability, but not necessarily activity, at high temperature, often by introduction of rigidifying salt-bridge interactions (Lam et al. 2011). Indeed, a comparison of primary structures of ycfD_RM_ and ycfD_EC_ (Fig. 1a) reveals a relative increase in highly charged, or rigidifying amino acids (relative abundance of Arg, Glu, Pro is increased by 4, 2 and 1%, respectively) and a decrease in residues with less-concentrated charge (relative abundance of Ser, Asp, Asn and Gln is decreased by 2, 3, 3 and 1%, respectively) in ycfD_RM_, compared to mesophilic ycfD_EC_.

Intact protein MS analysis revealed that uL16_RM_ hydroxylation levels decrease with increasing temperature. We, therefore, investigated what factors may limit the catalytic activity of ycfD_RM_. The results of thermal denaturation experiments, including DSF and CD, as well as MALDI–MS-based kinetic assays indicate that protein stability, as well as Fe(II) and 2OG availability (at least in vitro), are not likely to be limiting factors in ycfD_RM_ activity, even at relatively high temperatures.

We, therefore, addressed the dependence of ycfD_RM_ on molecular oxygen. With the caveat that we used a uL16_RM_ peptide fragment (20-mer) rather than intact protein, it is notable that the *K*_m_^app^ (O_2_) for ycfD_RM_ was 100 ± 20 μM. The *K*_m_^app^ for O_2_ is, therefore, apparently in the range of oxygen solubility limits (Tromans 1998). The rate of ycfD_RM_ catalysis is apparently linearly dependent on O_2_ availability; therefore, slight changes in O_2_ concentration will likely result in changes in ycfD_RM_ activity. This observation is consistent with the temperature-dependent linear decrease in levels of uL16_RM_ hydroxylation observed between 60 and 75 °C in *R.* *marinus* cells (Fig. 1d). Thus, O_2_-regulated changes in ycfD_RM_-catalysed uL16 hydroxylation have potential to be biologically relevant, potentially in a hypoxia sensing capacity. The latter is a possibility of interest given the roles of 2OG oxygenases in hypoxia sensing by animals (Hausinger and Schofield 2015; Schofield and Ratcliffe 2004). Interestingly, uL16 hydroxylation in *E. coli* is > 95% at 37 °C, the optimal growth temperature for this organism (Ge et al. 2012).

The identification of ycfD_RM_ as 2OG dependent extends the known range of these ubiquitous enzymes. The observation of its variable activity in cells raises the possibility that uL16 hydroxylation has a signalling or regulatory role. It is notable that hydroxylated ribosomal protein residue R82 on uL16 has been described as crucial for the process of translocation during peptidyl transfer (Bock et al. 2013), and that ycfD knock-out in *E. coli*, devoid of uL16 hydroxylation, leads to a lower rate of peptide synthesis (Ge et al. 2012). Future research can focus on further studies employing genetic methods to study the roles of ycfD_RM_ and uL16 hydroxylation in vivo in *R.* *marinus*, and more generally on the importance of ribosome hydroxylation and its possible roles in the regulation of protein synthesis, including at high temperatures and, potentially, in a hypoxia sensing capacity.

## Electronic supplementary material

Below is the link to the electronic supplementary material.

Supplementary material 1 (DOCX 480 kb)
