# Supplementary material for: YcfDRM is a thermophilic oxygen-dependent ribosomal protein uL16 oxygenase
Source: Extremophiles. 2018 Mar 9;22(3):553–62. doi: 10.1007/s00792-018-1016-9 (PMC5862935; doi:10.1007/s00792-018-1016-9)
Supplement: Supplementary file 1 — Supplementary material 1 (DOCX 480 kb) [file 792_2018_1016_MOESM1_ESM.docx]

**YcfD_RM_ is a thermophilic oxygen-dependent ribosomal protein uL16 oxygenase**

Rok Sekirnik,^1,2^ Sarah E. Wilkins,^1^ Jacob Bush,^1^ Hanna Tarhonskaya,^1,3^ Martin Münzel,^1,4^ Aayan Hussein,^1^ Emily Flashman,^1^ Shabaz Mohammed,^1^ Michael A. McDonough,^1^ Christoph Loenarz,^1,5^ and Christopher J. Schofield^1, §^

^1^Chemistry Research Laboratory, Department of Chemistry, University of Oxford, 12 Mansfield Road, Oxford, OX1 3TA, United Kingdom.

^2^Present address: Novartis Technical Operations – Biosimilars, BTDM Mengeš, Lek d.d., Kolodvorska 27, 1234-Mengeš, Slovenia.

^3^Present address: AbbVie Deutschland GmbH & Co. KG, Drug Product Development, Knollstraßem 67061 Ludwigshafen, Germany.

^4^Present address: Novo Nordisk A/S, Novo Nordisk Park, 2760 Måløv, Denmark.

^5^Present address: Institute of Pharmaceutical Sciences, Albert-Ludwigs-Universität Freiburg, 79104 Freiburg, Germany.

§To whom correspondence should be addressed: Christopher J. Schofield, Department of Chemistry, Oxford, OX1 3TA; Tel: +44(0)1865285006; E-mail: christopher.schofield@chem.ox.ac.uk

Table 1: ESI-TOF-MS deconvoluted masses of *R. marinus* 30S ribosomal proteins.

| **Protein** | **Sequence mass (Da)** | **Experimental mass (Da)** | **Δ (Da)** | **Methionine lost?** | **Δ NML (Da)** | **PTM** |
| --- | --- | --- | --- | --- | --- | --- |
| **bS1** | 80651.5 | n.d. | n.a. | n.a. | n.a. | n.a. |
| **uS2** | 31152.8 | n.d. | n.a. | n.a. | n.a. | n.a. |
| **uS3** | 28438.8 | n.d. | n.a. | n.a. | n.a. | n.a. |
| **uS4** | 23726.6 | 23597 | -129.6 | Yes | 1.6 |  |
| **uS5** | 19516.5 | 19384.9 | -131.6 | Yes | -0.4 |  |
| **bS6** | 17188.2 | 17056.8 | -131.4 | Yes | -0.2 |  |
| **uS7** | 17847.7 | 17847.3 | -0.4 | No | -0.4 |  |
| **uS8** | 14960.3 | 14828.8 | -131.5 | Yes | -0.3 |  |
| **uS9** | 15219.6 | 15160.1 | -59.5 | Yes | 72 | unassigned |
| **uS10** | 11500.5 | 11369 | -131.5 | Yes | -0.3 |  |
| **uS11** | 13979.9 | 13863.4 | -116.5 | Yes | 14.7 | +Me |
| **uS12** | 13757.1 | 13624.2 | -132.9 | Yes | -1.7 |  |
| **uS13** | 14462.9 | 14331.1 | -131.8 | Yes | -0.6 |  |
| **uS14** | 10454.4 | 10322.8 | -131.6 | Yes | -0.4 |  |
| **uS15** | 10671.4 | 10671 | -0.4 | No | -0.4 |  |
| **bS16** | 23179.8 | 23048.2 | -131.6 | Yes | -0.4 |  |
| **uS17** | 11986.9 | 11855.2 | -131.7 | Yes | -0.5 |  |
| **bS18** | 7595.9 | 7464 | -131.9 | Yes | -0.7 |  |
| **uS19** | 10891.7 | 10760.1 | -131.6 | Yes | -0.4 |  |
| **bS20** | 10008.8 | 9877.4 | -131.4 | Yes | -0.2 |  |
| **bS21** | 8251.6 | 8119.9 | -131.7 | Yes | -0.5 |  |

Column 1 lists the ribosomal proteins analyzed; Column 2 lists the calculated unmodified masses based on DNA sequence information; Column 3 lists the experimentally observed masses for individual proteins; Column 4 lists calculated differences between predicted and experimental masses; Column 5 indicates a prediction of *N*-terminal methionine loss (NML) (Yes if Δ(Da) is between 129-132 Da or a composite thereof; No otherwise); Column 6 indicates differences between predicted and observed masses after accounting for NML; Column 7 indicates predicted post-translational modifications based on the calculated mass differences.

Table 2: ESI-TOF-MS deconvoluted masses of *R. marinus* 50S ribosomal proteins.

| **Protein** | **Sequence mass**  **(Da)** | **Experimental**  **mass (Da)** | **Δ (Da)** | **Methionine**  **lost?** | **Δ NML (Da)** | **PTM** |
| --- | --- | --- | --- | --- | --- | --- |
| **uL1** | 25877.8 | 25746.1 | -131.7 | Yes | -0.5 |  |
| **uL2** | 30710.3 | 30579 | -131.3 | Yes | -0.1 |  |
| **uL3** | 22999.7 | 22868 | -131.7 | Yes | -0.5 |  |
| **uL4** | 24128.5 | 24127.5 | -1 | No | -1.0 |  |
| **uL5** | 21822.5 | 21690.5 | -132 | Yes | -0.8 |  |
| **uL6** | 20273.6 | 20142 | -131.6 | Yes | -0.4 |  |
| **bL9** | 18216 | n.d. | n.a. | n.a. | n.a. | n.a. |
| **uL10** | 18722.7 | 18591.5 | -131.2 | Yes | 0.0 |  |
| **uL11** | 15635.3 | 15631 | -4.3 | Yes | 126.9 | +9 Me |
| **bL12** | 12988.9 | 12857.09 | -131.8 | Yes | -0.6 |  |
| **uL13** | 16703.4 | 16703.1 | -0.3 | No | -0.3 |  |
| **uL14** | 13475.6 | 13475.2 | -0.4 | No | -0.4 |  |
| **uL15** | 16420.9 | 16420.5 | -0.4 | No | -0.4 |  |
| **uL16** | 16275.3 | 16306 | 30.7 | No | 0.7 | +Me, +OH |
| **bL17** | 24984.9 | 24969 | -15.9 | No | -0.1 |  |
| **uL18** | 13512.6 | 13512.1 | -0.5 | No | -0.5 |  |
| **bL19** | 14247.4 | 14115.9 | -131.5 | Yes | -0.3 |  |
| **bL20** | 13561.8 | 13430.5 | -131.3 | Yes | -0.1 |  |
| **bL21** | 13416.6 | 13416.7 | 0.1 | No | 0.1 |  |
| **uL22** | 13588.9 | 13588.6 | -0.3 | No | -0.3 |  |
| **uL23** | 11506.5 | 11374.9 | -131.6 | Yes | -0.4 |  |
| **uL24** | 13577.7 | 13446 | -131.7 | Yes | -0.5 |  |
| **bL25** | 22065.1 | n.d. | n.a. | n.a. | n.a. | n.a. |
| **bL27** | 9295.6 | 9164 | -131.6 | Yes | -0.4 |  |
| **bL28** | 9054.6 | 8923 | -131.6 | Yes | -0.4 |  |
| **uL29** | 8571 | 8570.6 | -0.4 | No | -0.4 |  |
| **uL30** | 7733.1 | 7601.6 | -131.5 | Yes | -0.3 |  |
| **bL31** | 9497.7 | 9497.4 | -0.3 | No | -0.3 |  |
| **bL32** | 7748.1 | 7612.7 | -135.4 | Yes | -4.2 |  |
| **bL33** | 6687.8 | 6556.2 | -131.6 | Yes | -0.4 |  |
| **bL34** | 6228.4 | 6090.7 | -137.7 | Yes | -6.5 |  |
| **bL35** | 7907.5 | 7775.9 | -131.6 | Yes | -0.4 |  |
| **bL36** | 4523.3 | n.d. | n.a. | n.a. | n.a. | n.a. |

Column 1 lists the ribosomal proteins analyzed; Column 2 lists predicted unmodified masses based on DNA sequence information; Column 3 lists the experimentally observed masses for individual proteins; Column 4 lists the calculated differences between predicted and experimental masses; Column 5 indicates a prediction of *N*-terminal methionine loss (NML) (Yes if Δ(Da) is between 129-132 Da or a composite thereof; No otherwise); Column 6 indicates differences between predicted and observed masses after accounting for NML; Column 7 indicates predicted post-translational modifications based on the calculated mass differences.


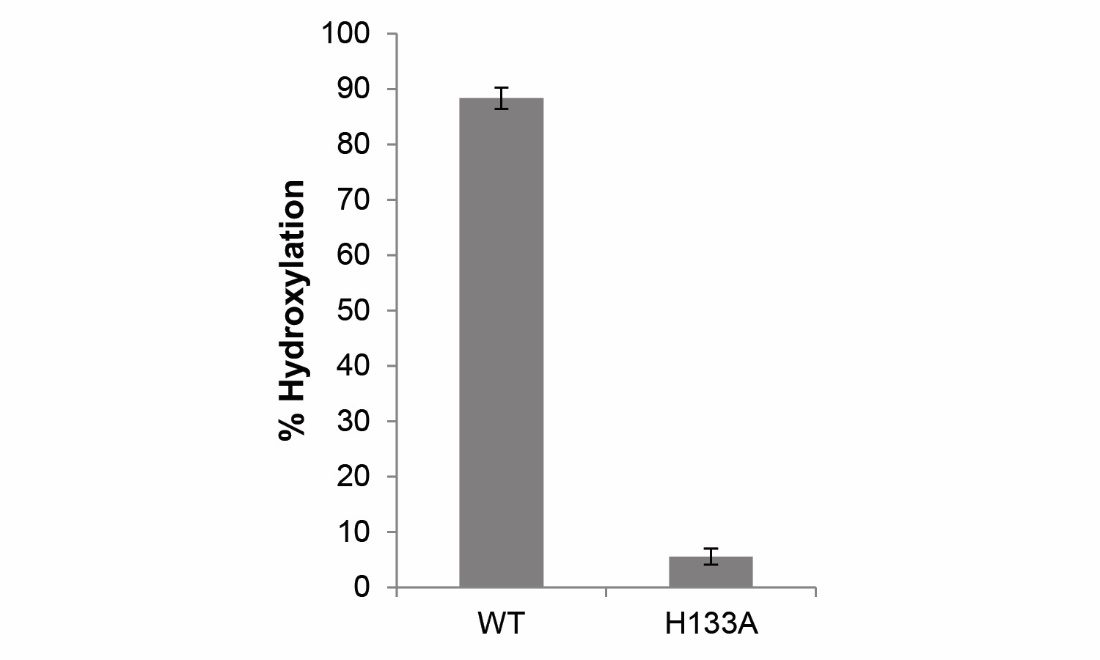


**Supplementary Figure 1: Activity of ycfD_RM_ WT and H133A Fe(II)-binding variant.** YcfD_RM_ WT or H133A (1 μM) were incubated with Fe(II) (as (NH_4_)_2_Fe(SO_4_)_2_, 100 μM), 2OG (as α-ketoglutaric acid (disodium salt dihydrate), 200 μM) and ascorbate (1 mM) in the presence of uL16_RM_ fragment peptide (NH_2_-KKPAEVRMGKGKGSVE (C-terminal amide), 100 μM) for 30 min at 70 ^o^C. Reactions were quenched with an equal volume of aqueous CF_3_COOH_aq_ (0.1% v/v) and the extent of hydroxylation analysed by MALDI-MS. The mean of three experiments is shown with error bars denoting standard deviation.


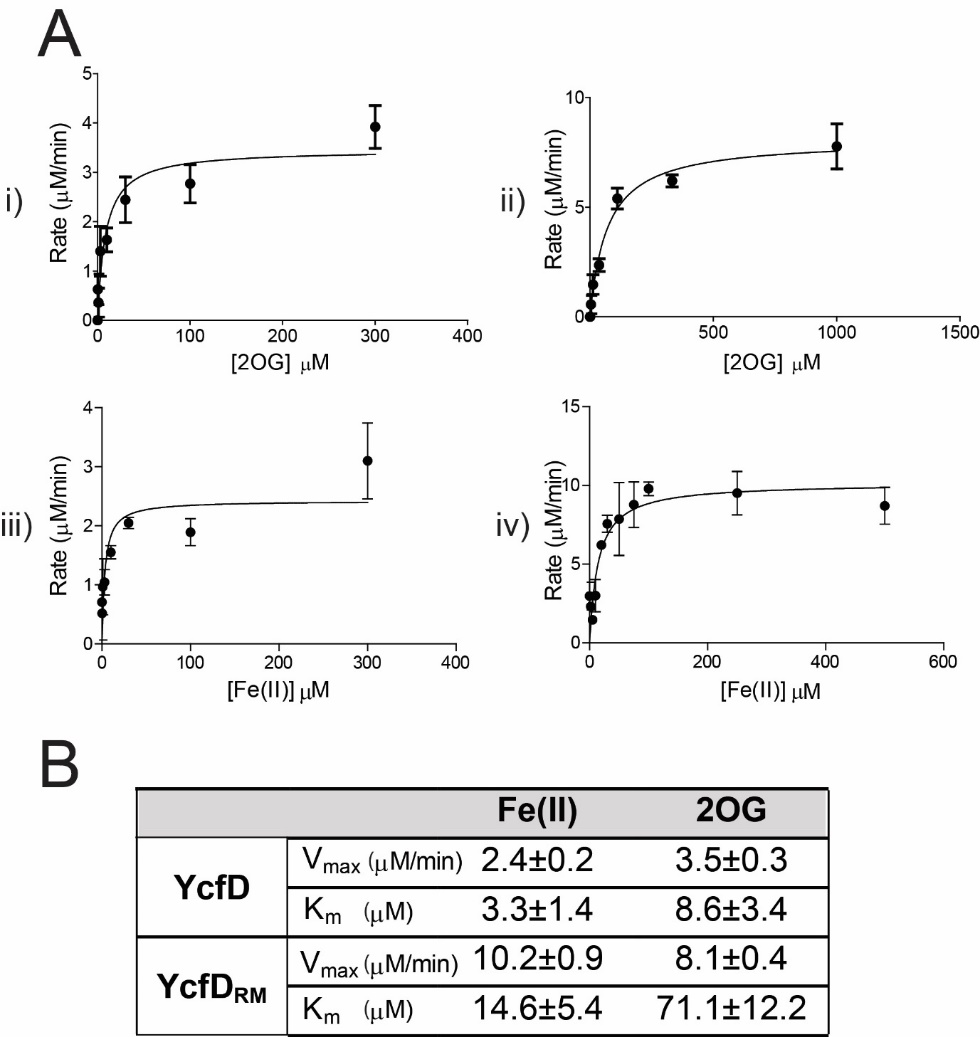


**Supplementary Figure 2: Rate-dependence of ycfD_EC_ and ycfD_RM_ on 2OG and Fe(II).** A: Rate-dependence on 2OG was determined for i) ycfD_EC_ and ii) ycfD_RM_ by incubation in the presence of serial dilutions of α-ketoglutaric acid (disodium salt dihydrate) and saturating concentrations of all other co-factors ((NH_4_)_2_Fe(SO_4_)_2_ at 100 μM, ascorbate at 1 mM, and substrate peptide (uL16_EC_ or uL16_RM_ for ycfD_EC_/_RM_, respectively at 400 μM in HEPES buffer (50 mM, pH 8.0/7.5 for ycfD_EC_/_RM_, respectively) at 37/55 ºC (ycfD_EC_/_RM_, respectively) and quenched after 1 min by addition of 1% CF_3_COOH_aq_. Rate-dependence on Fe(II) was determined for apo-forms of iii) ycfD_EC_ and iv) ycfD_RM_ (prepared by EDTA-treatment followed by buffer-exchange into reaction buffer) by incubation in the presence of serial dilutions of Fe(II) incubation and saturating concentrations of all other co-factors/co-substrates (ascorbate at 1 mM, 2OG at 200 μM and substrate peptide at 400 μM) in HEPES buffer (50 mM, pH 8.0/7.5 for ycfD_EC_/_RM_, respectively) at 37/55 ºC (ycfD_EC_/_RM_, respectively) and quenched after 1 min by addition of 1% CF_3_COOH_aq_. B: Kinetic parameters of ycfD_RM_ rate dependence on Fe(II) and 2OG concentrations.


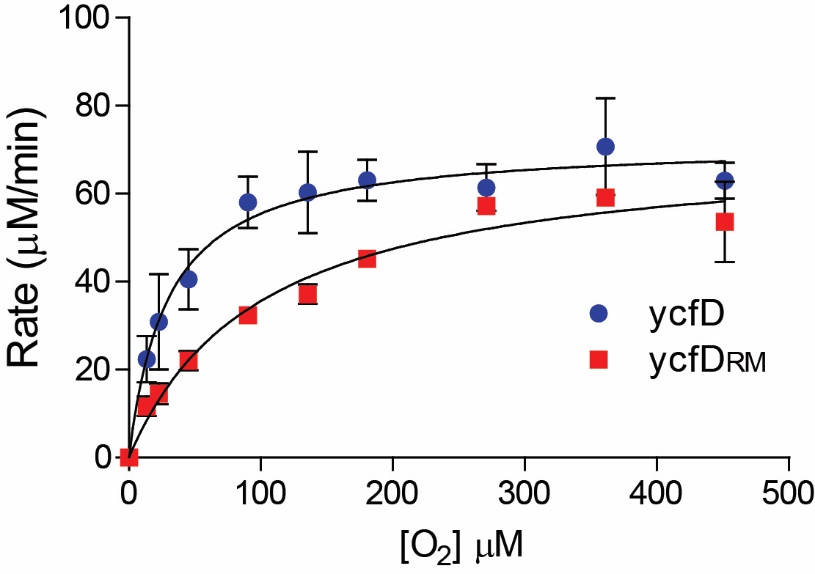


**Supplementary Figure 3**: **Steady-state kinetics of ycfD_RM_ and ycfD_EC_ dependence on O_2_**. The rate-dependence of ycfD_RM_ on O_2_ was determined by incubating ycfD_EC/RM_ (2 μM) with a solution of HEPES buffer (50 mM, pH 8.0/7.5 for ycfD_EC/RM_, respectively), Fe(II) at 100 μM, 2OG at 500 μM, ascorbate at 1 mM and uL16_EC/RM_ peptide fragment (ycfD_EC_ and ycfD_RM_, respectively) at 500 μM, separately pre-equilibrated at indicated concentrations of O_2_, controlled by a mass-flow controller. Reactions were carried out at 35 ºC and were quenched after 3 min with equal volume of CF_3_COOH_aq_ (1% v/v); conversion was monitored by MALDI-MS. Experiments were conducted in triplicate, with average values shown and error bars denoting standard deviation.


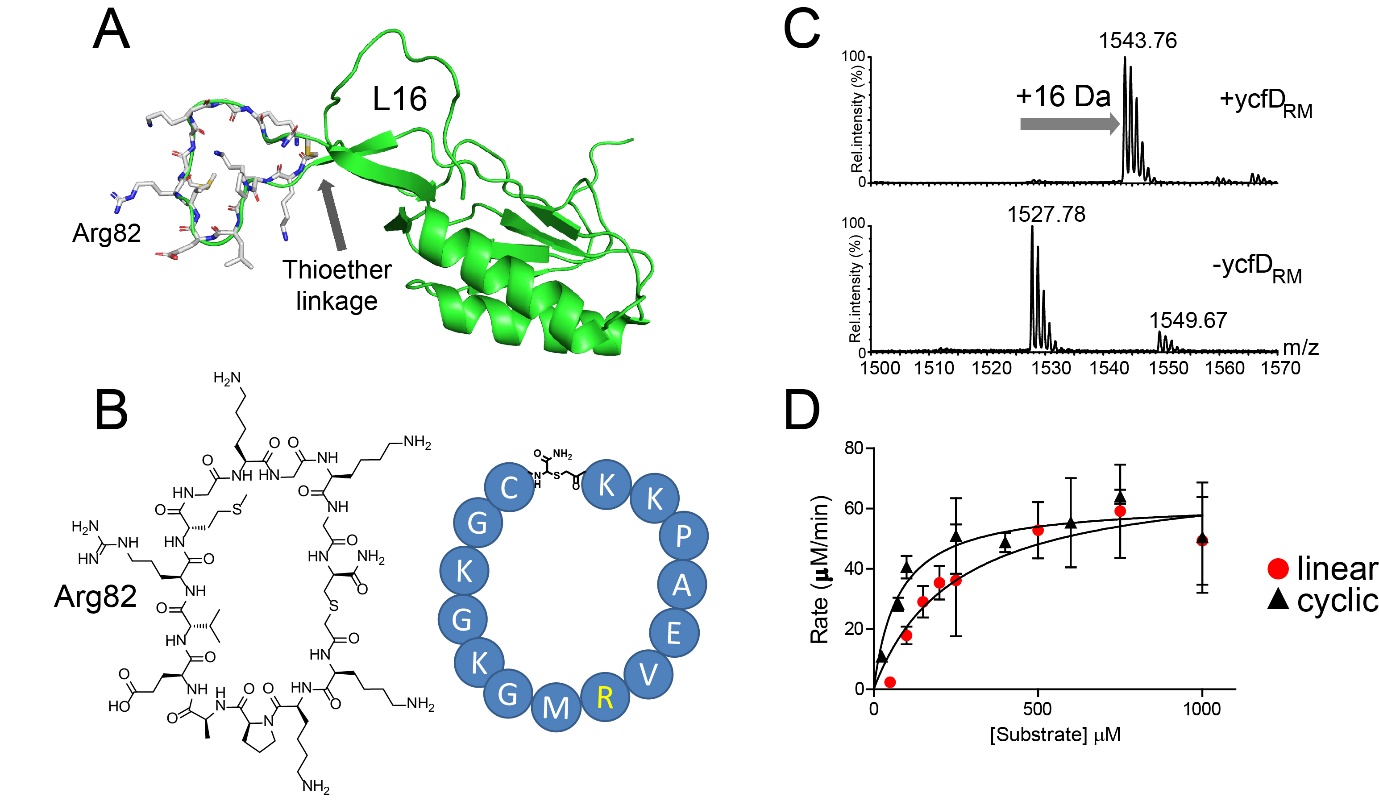


**Supplementary Figure 4: Cyclic uL16 fragment peptide increases the rate of hydroxylation.** A: Superposition of molecular model of the uL16_RM_-derived cyclic peptide and uL16 protein from crystal structure of *T. thermophilus* ribosomes (PDB: 2WDL). The peptide model was built in Pymol (DeLano Scientific, Version 1.3) using the “builder” tool. B: Structural formula and schematic representation of the synthesized uL16_RM_ cyclic peptide. C: MALDI-MS spectra of ycfD_RM_-dependent hydroxylation of uL16_RM_ cyclic peptide. The cyclic peptide was incubated with (top) or without (bottom) ycfD_RM_ (1 μM) in the presence of 2OG (200 μM), Fe2+ (100 μM) and ascorbate (1 mM) for 15 min at 55ºC, before quenching with an equal volume of CF_3_COOH_aq_ (1%) and analysed by MALDI-MS (calculated mass of uL16_RM_-cyclic peptide is 1528.8 Da). D: Kinetics of ycfD_RM_ with linear (KKPAEVRMGKGKGSVE, black triangles) or cyclic (KKPAEVRMGKGKGdC, red circles) uL16 fragment peptides.

**Supplementary Materials and Methods**

**Site-directed mutagenesis to prepare ycfD_RM__H133A**

Site directed mutagenesis of plasmid pET28 containing *ycfD_RM_* involved PCR and was conducted in a Techne Genius thermal cycler programmed for initial hold at 95 °C for 2 min before the addition of PfuTurbo® polymerase to the reaction mixture. This ensured denaturation of the double stranded DNA and prevented non-specific binding of primers. Reaction conditions and thermal cycling was according to manufacturer’s instructions.

The following primer pair was used to achieve the desired mutation:

| Forward primer | Reverse primer |
| --- | --- |
| GGGCGCATATCGCCAACTACGAC | ACGTCGTAGTTGGCGATATGCGC |

**Cyclic peptide modelling**

Models of cyclic peptides were built using Pymol (DeLano Scientific, Version 1.3) using the “builder” tool. The peptides were extracted and imported into ChemBio3D (Cambridgesoft, Version 12.0). The geometry was minimized using the “MM2” tool. The minimized molecules were re-imported into Pymol. Suitable cyclic peptide candidates were chosen after visual inspection.

**Cyclic Peptide Synthesis**

Amino acids for cyclic peptide synthesis were from CS Bio, Novabiochem, Sigma, TCI, Alfa Aesar, and AGTC Bioproducts. Peptides were analysed using an Agilent 1200 series LC-MS system (6120 quadrupole MS) with a Waters Sunfire column. Preparative HPLC purification was carried out on a Dionex Ultimate 3000 system with a Grace Vydac 218TP101522 column. Peptides were prepared as a *C*-terminal amide.

For the synthesis of the cyclic peptide a linear precursor was prepared by standard solid phase synthesis on a CS Bio CS336X peptide synthesizer (100 μmol scale) using *N,N'-*diisopropylcarbodiimide (DIC) as the coupling reagent. After cleavage of the *N*-terminal Fmoc-protecting group a solution of 150 mg of chloromethylcarbonyloxysuccinimide (ClAc-OSu) in 4 mL DMF was added to the resin and the mixture shaken for 3 h. The resin was filtered off and subsequently treated with 4 mL of deprotection solution (95 % (v/v) CF_3_COOH_aq_, 2.5 % triisopropylsilane, 2.5 % water). After 3 h the volume was reduced to 1 mL under a nitrogen stream and the peptides were precipitated with ice-cold Et­_2_O. The mixture was then centrifuged and the supernatant discarded. The solid was taken up in 1.5 mL of triethylammoniumacetate buffer (1 M, pH 8.5) and the pH readjusted to > 8 if necessary. In a microwave (Biotage Initiator) the mixture was heated to 80°C for 10 min and subsequently purified by HPLC (0-45 % MeCN in 45 min, 0.1 % CF_3_COOH_aq_, Dionex Ultimate 3000 series, Grace Vydac 218TP101522 column).

**Proteomic MS/MS analyses**

The *R. marinus* ribosomal protein samples digested with trypsin (37 ^o^C for either 15 min or overnight) were analyzed using an Orbitrap Elite (Thermo Fisher Scientific, DE) machine connected to a UHPLC Proxeon EASY-nLC 1000 and an EASY-Spray nano-electrospray ion source. Peptides were bound to an Acclaim PepMap® trapping column  (100 μm i.d. x 20 mm, 5 μm C18), then separated using an EASY-spray Acclaim PepMap® analytical column (75 μm i.d. x 500 mm, RSLC C18, 2 μm, 100 Å). Solvent A consisted of 0.1% formic acid and solvent B of 0.1% formic acid in acetonitrile. Peptides were separated using a 3 h gradient of 7% to 30% solvent B at a flow rate of 200 nL/min. Full scan MS spectra were acquired in the Orbitrap (350-1500 m/z, resolution 120,000, AGC target 1e6, maximum injection time 250 ms). CID and ETD spectra were acquired using the Ion Trap machine (resolution 7500, AGC cation target 3e4, AGC Anion target 2e5, maximum injection time 100 ms). The 20 most intense peaks in the full MS scan were selected for fragmentation using a data-dependent decision tree method (DDDT) (Swaney et al. 2008). ETD fragmentation was used for charge states 3, 4 and 5 with m/z less than 750 and for all charge states greater than 5. CID fragmentation was used for all other peptides.

 The raw data files generated were processed using MaxQuant software (Version 1.4.1.2), integrated with the Andromeda search engine (Cox et al. 2011). The maximum number of missed cleavages was set to be 4. Hydroxylation/oxidation of methionine-, lysine- and arginine-residues was allowed as a variable modification. All assignments of potential post-translational modifications were validated by manual inspection of spectra.

**Supplementary references**

Cox J, et al (2011), Andromeda: a peptide search engine integrated into the MaxQuant environment. J Proteome Res 4:1794-805.

Swaney DL, McAlister GC, Coon JJ (2008), Decision tree-driven tandem mass spectrometry for shotgun proteomics. Nat Methods 11:959-64.
